# Pharmacy Practice and Education in Bulgaria

**DOI:** 10.3390/pharmacy5030035

**Published:** 2017-06-22

**Authors:** Valentina Petkova, Jeffrey Atkinson

**Affiliations:** 1 Faculty of Pharmacy, Medical University-Sofia, 2-Dunav Street, 1000 Sofia, Bulgaria; petkovav1972@yahoo.com; 2 Pharmacolor Consultants Nancy, 12 rue de Versigny, 54600 Villers, France

**Keywords:** pharmacy, education, practice, Bulgaria

## Abstract

Pharmacies in Bulgaria have a monopoly on the dispensing of medicinal products that are authorized in the Republic of Bulgaria, as well as medical devices, food additives, cosmetics, and sanitary/hygienic articles. *Aptekari* (pharmacists) act as responsible pharmacists, pharmacy owners, and managers. They follow a five year Masters of Science in Pharmacy (M.Sc. Pharm.) degree course with a six month traineeship. *Pomoshnik-farmacevti* (assistant pharmacists) follow a three year degree with a six month traineeship. They can prepare medicines and dispense OTC medicines under the supervision of a pharmacist. The first and second year of the M.Sc. Pharm. degree are devoted to chemical sciences, mathematics, botany and medical sciences. Years three and four center on pharmaceutical technology, pharmacology, pharmacognosy, pharmaco-economics, and social pharmacy, while year five focuses on pharmaceutical care, patient counselling, pharmacotherapy, and medical sciences. A six month traineeship finishes the fifth year together with redaction of a master thesis, and the four state examinations with which university studies end. Industrial pharmacy and clinical (hospital) pharmacy practice are integrated disciplines in some Bulgarian higher education institutions such as the Faculty of Pharmacy of the Medical University of Sofia. Pharmacy practice and education in Bulgaria are organized in a fashion very similar to that in most member states of the European Union.

## 1. Introduction

Concerning general health in Bulgaria, the World Health Organization (WHO) estimated that a person born in Bulgaria in 2016 can expect to live 74.6 years on average: 78 years if female and 71.2 years if male (Table 1). WHO also estimated that life expectancy at birth for both sexes increased by 3 years over the period of 2000–2012; the WHO regional average increased by 4 years in the same period. In 2012, healthy life expectancy, in both sexes, was 9 years lower than the European average. This lost healthy life expectancy represents 9 equivalent years of full health lost through years lived with morbidity and disability.

Despite these somewhat disappointing figures, progress has been made in the past 30 years. Since the disruption of the established order of the Soviet Union in 1989, the on-going economic, political, and social changes in Bulgaria have had an important impact on all aspects of social life in the country, including pharmaceutical activities. Until 1989, the pharmaceutical system was centralized—community pharmacies, hospital pharmacies, wholesalers, pharmaceutical factories, and institutes were owned by the state. The importation and exportation of drugs were controlled by the state. 

Following the changes in 1989, the Bulgarian pharmaceutical system is oriented towards the private sector. Community pharmacies, wholesalers, and many drug manufacturers are now private entities. The first Bulgarian Law on drugs and pharmacies in human medicine was introduced in 1995 [2]. It lays out the structure for harmonization of Bulgarian drug regulatory affairs with those of the European Union. 

All these specific circumstances, together with a more global perspective on new drug discoveries and pharmaceutical technologies and methodologies, are a constant challenge leading to re-evaluation of the role of pharmacists in the Bulgarian health care system. Before these changes, a majority of Bulgarian pharmacists’ time was spent manufacturing drugs in the pharmacy. Nowadays, pharmacists apply different skills that require a detailed knowledge of communications and human behavior to scientifically dispense medications, and to counsel patients about their health and the correct use of their prescribed and OTC drugs. They are also responsible for monitoring patients to avoid adverse drug reactions and to achieve maximum benefit from the treatment. A very recent development is the implementation of the concept of “pharmaceutical care” as a central element of pharmacy practice. 

The Medical University in Sofia will be taken as an example for Bulgaria. The university has four faculties: medicine, dentistry, pharmacy, and social health. The pharmacy faculty is the oldest in Bulgaria in educating pharmaceutical specialists. The duration of the education is five years for community, hospital, and industrial pharmacists. All the graduates receive a M.Sc. Pharm. degree. One hundred to 120 Bulgarian and 80–100 foreign students are accepted for pharmacy education and training every year. 

There are six departments in the Faculty of Pharmacy in the Medical University in Sofia:

1. Pharmaceutical Technology and Bio-Pharmacy
2. Pharmacognosy and Pharmaceutical Botany
3. Pharmaceutical Chemistry
4. Chemistry
5. Pharmacology and Toxicology
6. Social Pharmacy

Following graduation, students have the opportunity to specialize for a further 3 years. Whilst working in a hospital or industrial environment, they follow a study program with courses at the faculty of pharmacy two weeks per year. After the third year of such specialization they pass a state examination in a given specialty. This possibility is granted by the Ministry of Education to all pharmaceutical students and graduates.

Since 1989, there have been many changes in the curriculum to harmonize courses and diplomas with those of the other schools in the European Union (EU). Many new study areas have been introduced such as: bio-pharmacy, clinical laboratory testing and analysis, and biology. The Department of Social Pharmacy has introduced new study areas such as: history of pharmacy, pharmaco-epidemiology, pharmaco-economics, pharmaceutical law, pharmaceutical marketing, and pharmaceutical management. 

In 2000, a new course in pharmaceutical care was introduced. The lectures and seminars on this subject are given during the first semester of the fifth year. The lectures synthesize the knowledge gained during the five-year pharmacy course and blend this with communication skills and the development of the logic of pharmaceutical care. University lecturers together with pharmacy practitioners, provide the training.

## 2. Design

Given the changes in pharmacy practice and education in Bulgaria outlined above, the PHARMINE (*Pharmacy Education in Europe*) European consortium surveyed the state of pharmacy education and practice in Bulgaria in 2012, with an update in 2017. The PHARMINE consortium was interested in general practice and education and in specialization in pharmacy education for hospital and industrial pharmacy practice. The survey also looked at the impact of the Bologna agreement on harmonization of the various European degree courses [3], and on the directive of the European Commission on education and training for the sectoral profession of pharmacy [4]. These two documents are somewhat contradictory in that the Bologna agreement proposes a bachelor plus master degree structure for all degrees including pharmacy, whereas as the European directive lays down a five-year “tunnel” degree structure for pharmacy, i.e., a degree course that has no possibility for intermediate entry or exit for example after a three-year bachelor period. The methodology used in the PHARMINE survey [5] and the principal results obtained in the EU [6] have already been published.

## 3. Evaluation and Assessment

### 3.1. Organisation of the Activities of Pharmacists, Professional Bodies

Table 2 provides details of the numbers and activities of community pharmacists and pharmacies in Bulgaria.

The data in Table 2 shows that compared to the EU linear regression estimation (for definition and calculation see reference 5) the ratio of the actual number of community pharmacists in Bulgaria (/population) compared to the linear regression estimation for Bulgaria = 1.16. Thus number of pharmacists per population is very close to the EU norm. The same comparison for community pharmacies produces a ratio of 1.99. Thus the number of community pharmacies in Bulgaria is double the EU average. 

The activities and occupations of pharmacists in Bulgaria are similar to those of community pharmacists in other member states [5]. The organization of community pharmacists regarding ownership, etc. is similar to that elsewhere in the EU; it should be noted that there are no government-imposed rules on the geographical distribution of community pharmacies in Bulgaria. The sale of medicinal products on the internet is limited to authorized pharmacies.

Table 3 provides details of the numbers and activities of assistant pharmacists in Bulgaria.

Bulgarian legislation recognizes that assistant pharmacists are health care professionals and defines their role in the health care system. Five pharmaceutical colleges provide education and training for assistant pharmacists. Although this is in the form of a three-year course, it cannot be compared to a “B. Pharm.” as defined by the Bologna declaration (see above).

Table 4 provides details of the numbers and activities of hospital pharmacists in Bulgaria.

Bulgarian legislation recognizes the existence of a hospital pharmacy, although the number of hospital pharmacists is low compared to the EU average. The ratio of the actual number of hospital pharmacists in Bulgaria (/population) compared to the linear regression estimation for Bulgaria = 0.29, (for definition and calculation see reference 5). The estimated number of hospital pharmacies is higher than that of hospital pharmacists. It appears therefore that the function of “hospital pharmacist” in Bulgaria is defined by competences and roles and/or by place of work. In the latter case, health care personnel other than pharmacists are involved.

Table 5 provides details of the numbers and activities of industrial pharmacists and pharmacists in other sectors, in Bulgaria.

Industrial pharmacists in Bulgaria have similar practices and duties to those in other EU countries [5]. As numbers of industrial pharmacists were not available for most European countries a comparison with the EU average is not possible.

Table 6 provides information on professional associations for pharmacists in Bulgaria.

The Bulgarian pharmaceutical union, which is the representative organisation of pharmacists in the country, oversees pharmacy education and training (PET), pharmacy practice, and ethics in a fashion similar to that in other member states of the EU [5].

### 3.2. Pharmacy Faculties, Students, and Courses

Table 7 provides details of pharmacy higher education institutions (HEIs), staff and students in Bulgaria.

The ratios of the actual number of HEIs, staff, and students in Bulgaria (/population) compared to the linear regression estimations for Bulgaria are 1.07, 1.01, and 0.76, respectively (for definition and calculation see reference [5]). Thus, figures for Bulgarian PET reflect those of the EU average for the country with a population the size of that of Bulgaria. Student numbers show a substantial international intake. It should be noted that the Erasmus Programme (European Region Action Scheme for the Mobility of University Students) is an EU student exchange program. Table 8 provides details of specialization electives in pharmacy HEIs.

Both pre- and post-graduate specialization are possible in Bulgaria. The last wave of pharmacists in post-graduate specialization in the medical university of Sofia was composed as follows—social pharmacy: 25; pharmacognosy: one; pharmaceutical analysis: one; pharmaceutical technology: one; industrial pharmacy: three. In this context, social pharmacy can be considered to consist of all the social factors that influence medicine use.

Table 9 provides details of past and present changes in education and training in Bulgarian pharmacy HEIs.

### 3.3. Teaching and Learning Methods

Table 10 provides details of student hours [18] by learning method. The data from Sofia is taken as an example in this table and Table 11.

Regarding the validation of traineeship, the pharmacist responsible for the trainee fills in a monthly and a final report at the end of the six months and these are validated (or not) by the HEI. It is to be noted that “practical” work is carried out by students at the university in the form of personnel projects, etc., whereas “traineeship” refers to work in a pharmacy setting.

### 3.4. Subject Areas.

Table 11 provides details of student hours by subject area.

### 3.5. Impact of the Bologna Principles [3]

Table 12 provides details the various ways in which the Bologna declaration impacts on Bulgarian pharmacy HEIs.

Data in the above table are in exchange months per year. The faculty of pharmacy in Sofia has ERASMUS exchange programs with:

○ Belgium, University of Antwerp and Vrije Universiteit Brussels
○ France, Université de Lorraine, Nancy and Université de Limoges
○ Germany, Ruprecht-Karls-Universität Heidelberg, Anhalt University of Applied Sciences Kothen and Freie Universität Berlin
○ Czech Republic—University of Veterinary and Pharmaceutical Sciences, Brno
○ Italy—Universita’ degli studi di Siena and Sapienza, University of Rome
○ Spain—University of Navarra and Universitat autonoma de Barcelona

There is also an exchange program with Turkey—Mersin University.

### 3.6. Impact of EU Directive 2013/55/EC 

Table 13 provides details the various ways in which the EC directive impacts on Bulgarian pharmacy HEIs [3].

Bulgarian PET mainly conforms to the different aspects of the EC directive with notably a five-year tunnel degree. Aspects of the Bologna agreement such as European Credit Transfer System (ECTS) and the Diploma Supplement are included.

**Figure 1:**
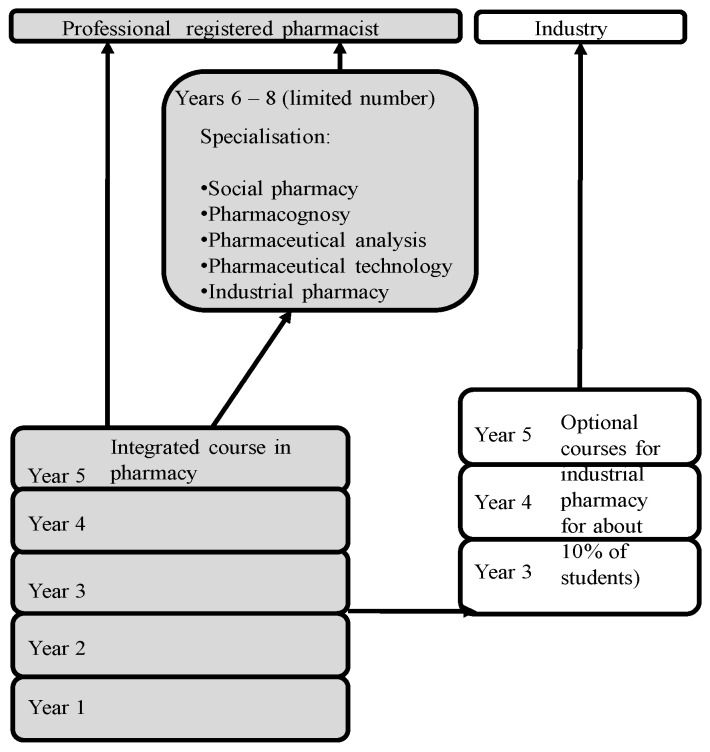
The scheme of pharmacy education and training (PET), in Bulgaria.

Figure 1 shows the scheme of PET in Bulgaria.

## 4. Discussion and Conclusions

Pharmacies in Bulgaria have a monopoly on the dispensing of medicinal products in Bulgaria. Pharmacists follow a five-year (M.Sc. Pharm.) degree course with a six months traineeship. The first and second year of the M.Sc. Pharm. degree are devoted to chemical sciences, mathematics, botany, and medical sciences. Years three and four center on pharmaceutical technology, pharmacology, pharmacognosy, pharmaco-economics, and social pharmacy, and year five on pharmaceutical care, patient counselling, pharmacotherapy, and medical sciences. A six month traineeship finishes the fifth year together with redaction of a master thesis, and the four state examinations with which university studies end. Industrial pharmacy and clinical (hospital) pharmacy practice are integrated disciplines in some Bulgarian HEIs, such as the Faculty of Pharmacy of the Medical University of Sofia. 

Following the changes in Bulgaria in 1989, pharmacy practice and education are organized in a fashion very similar to that in (most member states of) the European Union. Whilst new developments in pharmaceutical care with elements such as immunization, advice on tobacco use cessation, management of medication adherence, and provision of health screening to detect hypertension do not at the present time receive financial backing from the government, the fact that these elements are supported at the academic level, should reinforce the future role of the pharmacist in the promotion of patient well-being in Bulgaria.

## Figures and Tables

**Table 1:** Health statistics for Bulgaria [1].

Total Population	7,067,024
Life expectancy at birth m/f (years)	71.2/78 (2016)
Healthy life expectancy at birth m/f (years)	63/67 (2015)
Total expenditure on health per capita	1399$ (2014)
Total expenditure on health as % of GDP	8.4 (2014)

Statistics 30 January 2017 unless indicated.

**Table 2:** Numbers and activities of community pharmacists and pharmacies.

Item	Numbers	Comments
Pharmacists	5500–6000	1284 Inhabitants/Pharmacist
Pharmacies	4208	1.2–1.3 pharmacists per pharmacy 1679 inhabitants/pharmacy
Competences and roles of community pharmacists		After graduation from university, pharmacists can work in a pharmacy and can perform drug preparation, dispensing of drugs and consulting of patients on the proper drug treatment and prepare a pharmaceutical care plan (identifying drug-related problems, making a plan for proper drug treatment, monitoring of the treatment, etc.)
Is ownership of a community pharmacy limited to pharmacists?	No	The following are entitled to carry out retail trade in medicinal products: A natural or legal person. One who is registered as a pharmacy trader under the Bulgarian legislation or under the legislation of an EU member state. One who has signed a labour contract or a contract for management of a pharmacy with a pharmacist (in possession of an M.Sc. Pharm. degree. Or one who, in the cases provided under the law (no pharmacist available and until the arrival of master of a pharmacist), has signed a contract with an assistant pharmacist (for dispensation of OTC drugs only) One person may open no more than 4 pharmacies in Bulgaria [7].
Rules on geographical distribution of pharmacies?	No	There are no governmental restrictions on the geographical distribution of community pharmacies as a function of population density for instance.
Are drugs and healthcare products available to the general public by channels other than pharmacies?	No	Medicinal products, medical devices authorised in the Republic of Bulgaria, with or without medical prescription, as well as food additives, cosmetic, and sanitary-hygienic articles, are sold only in pharmacies. There are no mail-order pharmacies in Bulgaria. Any attempt to sell drug products at a lower price than originally planned is prohibited. Medicinal products not subject to a medical prescription may be sold on the internet only by a pharmacy or drugstore that has been granted authorisation under the terms and conditions of Medicinal Products in the Bulgarian Human Medicine Act [7].

**Table 3:** Numbers and activities of assistant pharmacists.

Are persons other than pharmacists involved in community practice?	Yes	In addition to pharmacists, assistant pharmacists are also considered to be professional pharmacy staff. Article 220/3 of the “Medicinal Products in Human Medicine Act” states that “An assistant pharmacist may carry out all operations under the control of a Master of Pharmacy, with the exception of: dispensation of a medicinal product under medical prescription, control, and consultations connected with medicinal products...” [7]. The assistant pharmacist´s code 5.7 states: “The students graduated from that speciality can work at the clinical pharmacy, at herbal stores, sanitary and drug stores, pharmacy stores, pharmacy laboratories, science institutes, and pharmaceutical factories.” [8].
Their titles and number(s)		There is no official data. There is no upper limit on the number; some pharmacies work without assistant pharmacists. There is a register of the pharmacists on the site of the Bulgarian Pharmaceutical Union [9]—but not of the assistant pharmacists.
Organizations providing and validating education and training of assistant pharmacists		Five pharmaceutical colleges provide education for assistant pharmacists: Medical College-Sofia: http://mu-sofia.bg/node/32 Medical College-Varna: http://www.mu-varna.bg/muVarna/index.php?option=com_content&task=view&id=193&Itemid=122 Medical College-Plovdiv: http://www.medcollege-plovdiv.org/ Medical College-Bourgas: http://www.btu.bg/bg/homebg.htm Pleven: http://www.mu-pleven.bg/index.php?lang=en&Itemid=254:
Duration of studies (years)	3	The studies of assistant pharmacists cannot be compared to bachelor studies at a university. There is no bachelor degree of “pharmaceutical education” in Bulgaria. There are uniform requirements for achievement of higher education as assistant pharmacist.
Conditions of entry		The entrance examination is in biology (that for pharmacy is in biology and chemistry). In some colleges there is also an interview.
Subject areas		Basic pharmaceutical sciences such as pharmaceutical chemistry, pharmaceutical technology, drug legislation, etc. The course lasts a minimum of 1200 h.
Competences and roles		Assist a pharmacist in the dispensation of OTC medicines only while under the supervision of a pharmacist.

**Table 4:** Numbers and activities of hospital pharmacists.

Does such a function exist?	Yes	The Bulgarian branch of the European Association of Hospital Pharmacists is the professional organization of the Bulgarian hospital pharmacies [10].
Number of hospital pharmacists	197	This is the number of pharmacists registered with the Bulgarian Association of Hospital Pharmacists [11]
Number of hospital pharmacies		There are 344 (2011) hospitals in Bulgaria—most have a hospital pharmacy.
Competences and roles of hospital pharmacists		Preparation and dispensing of drugs on hospital wards and also: Part of multidisciplinary patient-care team. Purchasing of drugs and medical material. Monitoring of drug use. Production of patient-specific medicines. Participation in clinical studies.

**Table 5:** Numbers and activities of industrial pharmacists and pharmacists in other sectors.

**Industrial Pharmacy and Pharmacists**		
Number of pharmaceutical companies with production, R&D and distribution	22	The European Federation of Pharmaceutical Industries and Associations (EFPIA) has 22 members in Bulgaria [12]. The Bulgarian representative is the Association of the Research-based Pharmaceutical Manufacturers in Bulgaria [13].
Number of companies producing generic drugs only	9	Examples: Actavis http://www.actavis.bg/bg/default.htm Sopharma http://www.sopharma.bg/
Number of pharmacists working in industry	About 1000	The number is estimated from the number of students graduating with the industrial pharmacy degree option; students taking the industrial pharmacy option account for <10% of the class size. EFPIA has estimated that the total number of people employed in the pharmaceutical industry equals 9900 [14].
Competences and roles		Drug manufacturing, control, analysis, registration, etc.
**Pharmacists Working in Other Sectors**		
Sectors in which pharmacists are employed		Academia (faculties of pharmacy) Wholesale Medical and pharmaceutical information Bulgarian Drug Agency Ministry of health Representative offices of Bulgarian and foreign drug companies Drug manufacturing in the Bulgarian drug companies.
Competences and roles in other sectors		Teaching, tutoring, drug accounting, communication, advertising, etc. The exact number of pharmacists working in other sectors in Bulgaria is impossible to determine.

**Table 6:** Professional associations for pharmacists in Bulgaria.

Registration of pharmacists	Yes	The Bulgarian Pharmaceutical Union [15] provides a certificate of entry onto the register of the corresponding Regional College of the Bulgarian Pharmaceutical Union, to every Master of Pharmacy who is at the head of a pharmacy. In order to be registered as a professional pharmacist one has to submit to the Bulgarian Pharmaceutical Union: Diploma of a higher educational pharmaceutical department. Diploma(s) for specialization (hospital, industrial) or Ph.D./DSc/Associate professor/Professor. Certificate from the working place attesting that he/she is working as a pharmacist. A certificate showing no previous criminal conviction. After approval, the pharmacist becomes a member of the Bulgarian Pharmaceutical Union and gains his/her unique identification number as a pharmacist.
Creation of pharmacies and control of territorial distribution	Yes	The Bulgarian Drug Agency issues an authorisation for retail trade in medicinal products in a pharmacy and controls the implementation of requirements for the retail trade of medicines.
Ethical and other aspects of professional conduct	Yes	The Bulgarian Pharmaceutical Union has an ethical code for pharmacy practice.
Quality assurance and validation of university courses	Yes	University courses are controlled by the quality commission of the Bulgarian Pharmaceutical Union [16]. http://bphu.eu/

**Table 7:** Pharmacy higher education institutions (HEIs), staff, and students in Bulgaria.

Item	Number	Comments			
Number of pharmacy HEIs in Bulgaria	5	Pharmacy HEIs: Medical University of Sofia : www.pharmfac.net University of Plovdiv: http://meduniversity-plovdiv.bg/index.php?lang_id=2&prm=fac&subprm=farf University of Varna: http://www.mu-varna.bg/ (started accepting students in 2009) Sofia University: http://www.uni-sofia.bg/index.php/eng/faculties/faculty_of_chemistry_and_pharmacy Medical University of Pleven: http://www.mu-pleven.bg/index.php/structure/faculty-of-pharmacy?lang=en			
Public pharmacy HEIs	5	There are no private pharmacy HEIs in Bulgaria.			
Faculty attachment		The faculties of pharmacy in Sofia (Medical University of Sofia), Plovdiv, Pleven and Varna are faculties of the corresponding medical universities. The faculty of Chemistry and Pharmacy (number 4 above) is part of Sofia University.			
Do HEIs offer B and M degrees?	No	Only an M.Sc. Pharm. Degree is offered; there is no Bulgarian B. Pharm degree (see later).			
**Teaching staff**					
Staff (nationals)	250				
Professionals from outside the HEIs	20	They are from the pharmacies (supervision of student traineeships), pharmaceutical companies, wholesalers, etc.			
**Students**					
Graduates that become registered pharmacists.	More than 400 per year	The number of graduates during the past five years was increased due to the increase in the number of the faculties and the introduction of a pharmacy course in English in most of the faculties—especially Sofia and Plovdiv.			
Number of places on entry following secondary school	260+ per year	For 2012 [ 17]: Medical university of Sofia: 120 Plovdiv: 60 Varna: 30 Sofia University: 50 Pleven: not available			
Number of applicants for each entry place		Medical University of Sofia: 3.4 Plovdiv: 1.8 Figures from reference 18.			
Number of non EU international students	≥ 50 per year	Mainly from Macedonia, Turkey, Morocco, Tunisia and Serbia.			
**Entry requirements following secondary school**					
Specific national entrance examination for pharmacy			Yes	National entrance examination in biology and chemistry.	
Is there a national *numerus clausus*?			No		
**Fees per year**					
For home students					375€
For EU MS students					375€
For non EU students					7000€

**Table 8:** Specialization electives in pharmacy HEIs.

Do HEIs Provide Specialized Courses?	Yes	Comments
In which years?	third, fourth and fifth; also post-graduate	
In which specialisation (industry, hospital…)?		Industry and clinical pharmacy after the third year.
What are the student numbers in each specialization?	15 (industry) and 12 (clinical pharmacy)/year for pre-graduate	Following graduation there is a possibility to start post-graduate specialization (three year course) in one of five different areas: industrial pharmacy; social pharmacy; pharmacognosy; pharmaceutical analysis; and pharmaceutical technology.

**Table 9:** Past and present changes in education and training in Bulgarian pharmacy HEIs.

Have there been any major changes since 1999?	Yes	The main changes were towards harmonising with the EU requirements—more practical than theoretical subjects. Teaching of “new” subjects such as, pharmaceutical care, pharmaco-economics, bromatology/food science, history of pharmacy, etc. Changes were made in the state exam in order to harmonize the final examinations to those of EU HEIs.
Are any major changes envisaged before 2019?	Yes	Changes in the relative number of hours of some subject areas. Chemical subjects will decrease while the special subjects like pharmaceutical technology will increase their number of hours.

**Table 10:** Student hours by learning method.

Method	Year 1	Year 2	Year 3	Year 4	Year 5	Total
Lecture	210	315	330	435	210	1500
Practical	540	525	585	825	345	2820
Hospital/community traineeship					800	800
Electives			90	120		
Total	750	840	915	1260	1355	5120

**Table 11:** Student hours by subject area (for definition of subject areas see [4]). The numbers are calculated according to the schema of the Uniform State Requirements of Bulgaria [14].

Subject Area	Year 1	Year 2	Year 3	Year 4	Year 5	Total
CHEMSCI	165	510	225	225	150	1275
PHYSMATH	300					300
BIOLSCI	60	165	75	150		450
PHARMTECH			210	315		525
MEDISCI	45	120		690	120	975
LAWSOC	30		90	45	120	285
GENERIC	300					300
GENERIC + TRAINEESHIP	300				800	800
Total	900	795	600	1425	1190	4910

CHEMSOC: chemical sciences; PHYSMATH: physical and mathematical sciences; BIOLSCI: biological sciences; PHARMTECH: pharmaceutical technology; MEDISCI: medicinal sciences; LAWSOC: law and social sciences; GENERIC: generic competences. Taking the MEDISCI/CHEMSCI ratio as an indicator [19] of the nature of the M. Pharm. degree course (ratio = 975/1,275 = 0.8) it appears that the Bulgarian course is more a “chemical science” course similar to that in Germany (ratio = 0.7), but different from “medicinal science” course given in Ireland (ratio = 2.6) [18].

**Table 12:** Ways in which the Bologna declaration impacts on Bulgarian pharmacy HEIs.

“Comparable degrees with diploma supplement”	Yes	The comparability of degrees is achieved through calculation of the hours and comparison with other EU countries. The Diploma Supplement provided is in English. The Diploma Supplement describes the nature, level, context, content, and status of the studies that were pursued. With the texts of the Law on Higher Education adopted by the Bulgarian Parliament on 4 June 2004 both the system for collection and transfer of credits and the Diploma Supplement were legally introduced.
“Two main cycles (B and M) with entry and exit at B level”	No	There is a five-year “tunnel” degree structure.
“European Credit Transfer System (ECTS) system of credits with links to life-long learning (LLL)”	Yes	The ECTS system of credits is applied during the fiv year period of learning and after graduation in the different courses of LLL.
“Addressing obstacles to mobility”	Partial	As the English language is not used extensively in Bulgaria there are language barriers for the proper application of mobility. Financial problems also exist.
“Application of European QA”	Partial	PET is regulated at a national level by the ministry of education, but it is harmonized to EU requirements
ERASMUS staff exchange to Sofia from elsewhere		Staff months: zero
ERASMUS staff exchange from Sofia to other HEIs		Staff months: one
ERASMUS student exchange to Sofia from elsewhere		Student months: 28
ERASMUS student exchange from Sofia to other HEIs		Student months: 72

**Table 13:** Ways (right column) in which the elements of the EC directive (left column) impact on Bulgarian pharmacy HEIs.

“Evidence of formal qualifications as a pharmacist shall attest to training of at least five years‘ duration,…”	The training of pharmacists M.Sc. in Bulgaria is five years induration. The curriculum covers the EU requirements.
“…four years of full-time theoretical and practical training at a university or at a higher institute of a level recognised as equivalent, or under the supervision of a university;”	Bulgaria complies.
“…six-month traineeship in a pharmacy which is open to the public or in a hospital, under the supervision of that hospital's pharmaceutical department.”	Bulgaria complies.
